# The Clinical Characteristics and Outcomes of Acute Pancreatitis Are Different in Elderly Patients: A Single-Center Study over a 6-Year Period

**DOI:** 10.3390/jcm13164829

**Published:** 2024-08-16

**Authors:** Shihang Zhang, Zhiyao Chen, Cheng Hu, Ping Zhu, Tao Jin, Lan Li, Ziqi Lin, Na Shi, Xiaoxin Zhang, Qing Xia, Lihui Deng

**Affiliations:** West China Centre of Excellence for Pancreatitis, Institute of Integrated Traditional Chinese and Western Medicine, West China Hospital, Sichuan University, Chengdu 610041, China; 2022224020188@stu.scu.edu.cn (S.Z.); 2019324020149@stu.scu.edu.cn (Z.C.); hucheng@wchscu.cn (C.H.); zhuping@wchscu.cn (P.Z.); jintao@wchscu.cn (T.J.); lilan@wchscu.cn (L.L.); linziqi@wchscu.cn (Z.L.); nashi@scu.edu.cn (N.S.); zhangxiaoxin@wchscu.cn (X.Z.)

**Keywords:** acute pancreatitis, elderly, clinical characteristics, outcomes

## Abstract

**Objectives:** This study aims to analyze the clinical characteristics of elderly patients with acute pancreatitis (AP) and investigate the effects of age on the clinical outcomes of AP. **Methods:** Patients aged ≥ 18 years with AP admitted within 72 h from 1 September 2013 to 31 August 2019 were included. Patients were divided into elderly (≥60 years) and non-elderly (<60 years) groups. Clinical data and outcomes were compared. **Results:** A total of 756 elderly and 4896 non-elderly patients with AP were included. The elderly patients had different etiological distributions and more severe clinical markers and scores. Age was an independent risk factor for mortality [odds ratio (OR): 2.911, 95% CI: 1.801–4.706, *p* < 0.001], intensive care unit admission (OR: 1.739, 95% CI: 1.126–2.685, *p* = 0.013), persistent organ failure (OR: 1.623, 95% CI: 1.326–1.987, *p* < 0.001), multiple organ failure (OR: 1.757, 95% CI: 1.186–2.604, *p* = 0.005), and infection (OR: 2.451, 95% CI: 1.994–3.013, *p* < 0.001). Adjusted multiple logistic regression and trend analysis confirmed the risk of the age for the outcomes. The deaths of elderly patients showed a biphasic pattern with peaks in the first and fifth weeks, in contrast to the single peak in the first week in the non-elderly patients. **Conclusions:** Elderly patients with AP were associated with worse clinical outcomes. It is crucial to devote considerable attention to the optimization of therapeutic approaches to reduce late mortality in this group of patients.

## 1. Introduction

Acute pancreatitis (AP) is an inflammatory disease of the pancreas associated with significant morbidity and mortality. It is triggered by the premature activation of trypsinogens and organelle dysfunction within pancreatic acinar cells, driven by cell death and local and systemic inflammation [1]. Epidemiological evidence indicates that the global incidence of AP is 34 per 100,000 person-years, with a continued upward trend [2,3,4]. Over the past decade, the overall mortality of AP has declined to approximately 2% due to improvements in timely and accurate diagnosis and treatment. However, the mortality and financial burden of severe acute pancreatitis (SAP) remain substantial.

In recent years, global life expectancy has increased, leading to a notable rise in the proportion of elderly individuals. Numerous health challenges are becoming more prevalent among the elderly, including an increased susceptibility to AP [5]. The global incidence of mortality due to pancreatitis in the elderly is rising and the burden of pancreatitis in this demographic is higher in less developed regions [6]. Studies indicate that elderly patients with AP, particularly those over 80 years old, experience more severe illness and are more prone to developing systemic complications, resulting in a higher mortality rate [7,8,9,10,11,12,13,14,15]. Previous studies have suggested that aging may exacerbate AP through immune system compromise and increased inflammation [5,16]. Nevertheless, some studies have indicated that age does not significantly affect the outcomes of AP [17,18,19,20]. In light of the lack of consensus regarding the precise impact of age on AP outcomes, it is imperative to devote greater attention to the prevention and the treatment of AP in the elderly [21].

In this study, we performed a cohort study using a prospectively collected database to determine the clinical characteristics of AP in elderly patients and the impact of age on the clinical outcomes of AP.

## 2. Methods

### 2.1. Study Patients and Grouping

Data were obtained from a prospective database of consecutive cohorts of patients with AP admitted to West China Hospital of Sichuan University. Patients were enrolled if they were over 18 years old and admitted within 72 h from the onset of abdominal pain between 1 September 2013 and 31 August 2019. Exclusion criteria included the following: (1) pregnant or lactating women; (2) patients with a history of any tumor diseases; and (3) patients in the advanced or terminal stage of any pre-existing disease.

According to the World Report on Ageing and Health by the World Health Organization (https://www.who.int/news-room/fact-sheets/detail/ageing-and-health (accessed on 12 May 2024)), older age was defined as patients aged ≥ 60 years. In this study, the patients were divided into the elderly (aged ≥ 60 years) and non-elderly (aged < 60 years) groups.

### 2.2. Clinical Data Collection

Demographic and clinical data were recorded prospectively in the database. Demographic characteristics included age, sex, onset hours, Charlson Comorbidity Index (CCI), American Analgesic Association (ASA) classification, etiology, referral, disease history of AP, comorbidities, laboratory variables, and clinical severity scoring systems including systemic inflammatory response syndrome (SIRS), modified Glasgow Coma Scale (GCS) scores, and Sequential Organ Failure Assessment (SOFA). Computed tomography (CT) images were accessed in the medical record system and the Modified Computed Tomography Severity Index (MCTSI) was independently assessed by two radiologists.

The diagnosis and severity classification was conducted in accordance with the revised Atlanta classification [22]. The management of patients with AP was based on the International Association of Pancreatology and the American Pancreatic Association Guidelines [23,24]. Patients at high risk of severe acute pancreatitis were admitted to the high dependency unit (HDU). Patients were transferred to the intensive care unit (ICU) if they were extubated and required invasive ventilation due to refractory organ failure. A step-up strategy was adopted for infected pancreatic necrosis (IPN) and surgery was performed if the patients’ illnesses were combined with failed peripancreatic drainage, pancreatic dissection, uncontrolled gastrointestinal or abdominal bleeding, and fistulas. For patients with the mild illness of acute biliary pancreatitis, cholecystectomy was suggested and performed during the initial admission [24].

### 2.3. Clinical Outcome Measures

Patients were followed up until discharge or death. The primary outcomes of this study included mortality. Secondary outcomes included organ failure, multiple organ failure (MOF), admission to HDU/ICU, invasive mechanical ventilation (IMV), local complications, necrosectomy, length of hospital stay, and overall mortality followed up to 3 months after hospital discharge.

### 2.4. Statistical Analysis

Continuous data were presented as medians with interquartile ranges (IQRs) and compared using Mann–Whitney U-tests. Categorical data were expressed as numbers and percentages, and the results were compared using chi-square tests or Fisher’s exact tests. Baseline variables were compared between groups using univariate analysis. Kaplan–Meier survival analyses and log-rank tests were performed to compare the prognosis of elderly patients and non-elderly patients. Multivariate logistic regression analysis was conducted to report categorical outcome measures, expressed as odds ratios (ORs) with 95% confidence intervals (CIs). In our previous study [25], multivariate logistic regression, the baseline covariates of clinical significance including gender, CCI, comorbidity, referral status, onset hours, and admission hypertriglyceridemia (HTG) were adjusted for the comparison of outcomes. A two-sided *p*-value of less than 0.05 was considered statistically significant. Statistical analyses were conducted using R version 4.2.0 (R Foundation for Statistical Computing, Vienna, Austria) and SPSS^®^ version 26.0 (IBM Corp., Armonk, NY, USA).

## 3. Results

### 3.1. Baseline Characteristics of Patients

**Figure 1:**
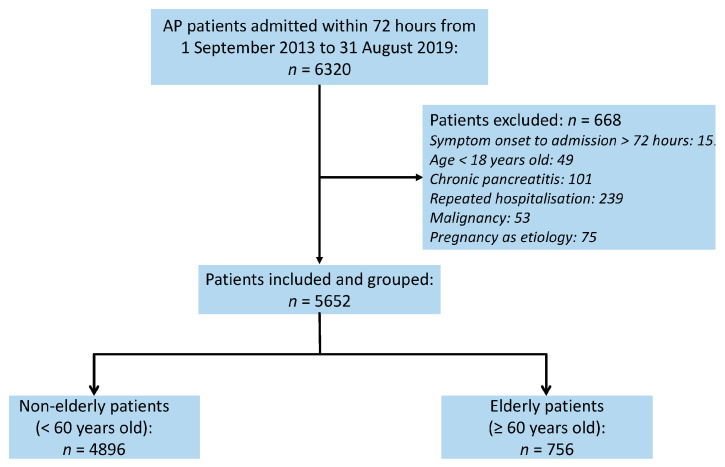
Flow chart of patient selection. AP, acute pancreatitis.

During the study period, 6320 patients were initially screened from the database, and 5652 patients were enrolled for analysis. The patient selection process is shown in Figure 1.

**Figure 2:**
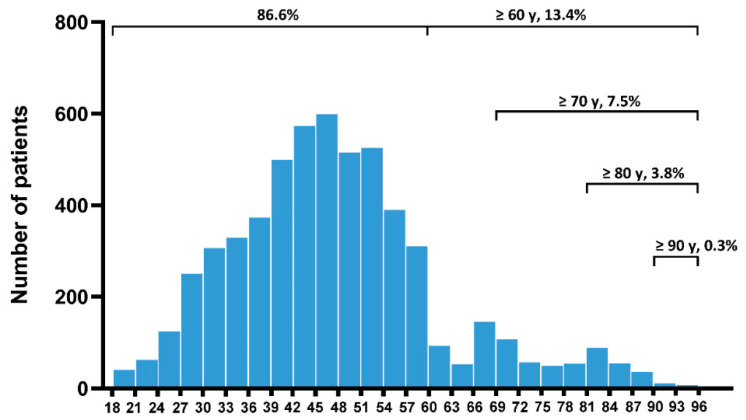
Distribution of age in the study cohort.

As shown in Figure 2, there were 4896 (86.6%) young and middle-aged patients (<60 years), and 756 (13.4%) elderly patients (≥60 years). A significant reduction was observed among patients aged > 60 years, with the highest proportion in the 60 to 69 age group. Significant differences were found in gender, CCI, etiology, recurrent AP, ASA, comorbidity, and vital signs between the two groups. Elderly patients had higher levels of blood urea nitrogen, creatinine, and interleukin 6 and increased clinical severity scores, while the levels of hematocrit, albumin, triglycerides, and C-reactive protein were lower than those of non-elderly patients. These findings indicate that elderly patients with AP present with more severe illness on admission (Table 1).

### 3.2. Clinical Outcomes of the Elderly Patients

As presented in Table 2, elderly patients had a higher mortality rate (4.0% vs. 1.6%, *p* < 0.001), a longer length of hospital stays (12 days vs. 9 days, *p* < 0.001) and ICU stay (0 day vs. 0 day, *p* = 0.040), and higher hospital costs (¥21,375.1 vs. ¥14,455.5, *p* < 0.001). Furthermore, there was an elevated incidence of persistent respiratory (23.7% vs. 17.9%, *p* < 0.001) and cardiovascular failure (4.5% vs. 2.9%, *p* = 0.015). Additionally, there were more cases of SAP (25.4% vs. 18.5, *p* < 0.001), a higher incidence of cholecystectomy (10.6% vs. 4.4%, *p* < 0.001), and a higher rate of infection in the lung (19.8% vs. 8.6%, *p* < 0.001) and blood (8.5% vs. 4.2%, *p* < 0.001). The utilization of non-invasive positive pressure ventilation (NIPPV) (17.2% vs. 13.6%, *p* = 0.009) and IMV (6.6% vs. 4.7%, *p* = 0.024) were higher in elderly patients. Conversely, elderly patients exhibited a reduced incidence of local complications, including acute peripancreatic fluid collection (34.4% vs. 41.0%, *p* < 0.001) and acute necrotic collection (6.6% vs. 10.0%, *p* = 0.003). After adjusting for baseline parameters, age was identified to be an independent risk factor for mortality (OR: 2.911, 95% CI: 1.801–4.706, *p* < 0.001), ICU admission (OR: 1.739, 95% CI: 1.126–2.685, *p* = 0.013), persistent organ failure (POF) (OR: 1.623, 95% CI: 1.326–1.987, *p* < 0.001), MOF (OR: 1.757, 95% CI: 1.186–2.604, *p* = 0.005), and infection (OR: 2.451, 95% CI: 1.994–3.013, *p* < 0.001) (Table 3).

### 3.3. Multivariate Logistic Regression

A multivariate logistic regression analysis was performed to verify whether age was an independent risk factor for mortality, ICU admission, infection, MOF, and POF (Table 4). The results showed that age > 60 years old, CCI > 2, referral status, and admission HTG ≥ 500 mg/dL were the independent risk factors for mortality. Age > 60 years old, male, referral status, onset hours ≥ 24 h, and admission HTG ≥ 500 mg/dL were the independent risk factors for ICU admission. Infections were significantly influenced by age > 60 years old, CCI > 2, referral status, and onset hours ≥ 24 h. Age > 60 years old, CCI > 2, referral status, onset hours ≥ 24 h, and admission HTG ≥ 500 mg/d were independent risk factors for MOF. Age > 60 years old, CCI > 2, comorbidity, referral status, onset hours ≥ 24 h, and admission HTG ≥ 500 mg/dL were independent risk factors for POF. These results emphasize that age is an independent risk factor for adverse outcomes in AP.

### 3.4. Trend Analysis for the Age and Outcomes in the Elderly Patients

**Figure 3:**
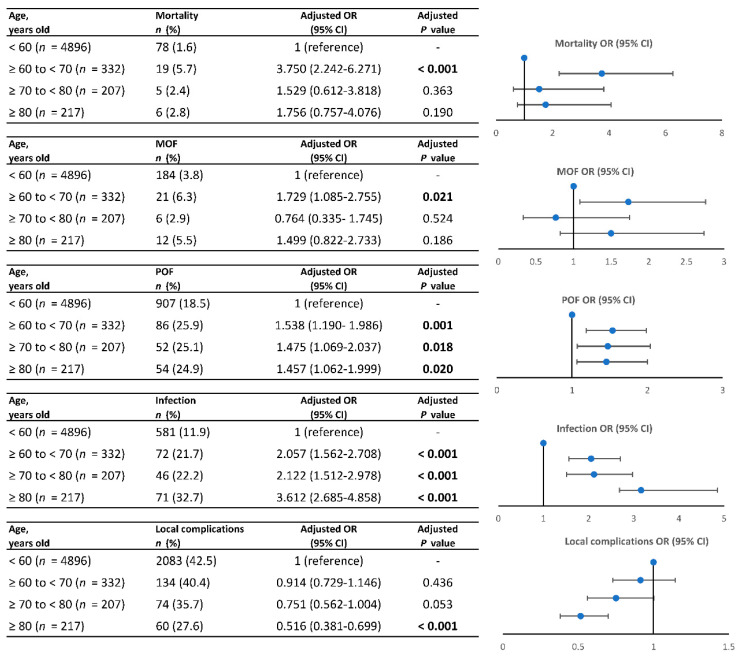
Trend analysis for age and outcomes in elderly patients. OR, odds ratio; MOF, multiple organ failure; POF, persistent organ failure. The blue spots represent the adjusted OR.

Trend analysis was conducted based on different age ranges, highlighting the impact of age on various clinical outcomes (Figure 3). In comparison to non-elderly patients, those aged 60 to 70 years had a significantly increased risk of mortality (adjusted OR: 3.750; 95% CI: 2.242–6.271) and MOF (adjusted OR: 1.729; 95% CI: 1.085–2.755). The adjusted ORs for POF showed a decreasing trend with the increase in age. Notably, there was an obvious deterioration in infection with increasing age, while the risk of local complications decreased with age.

### 3.5. Pattern of Mortality over Time

**Figure 4:**
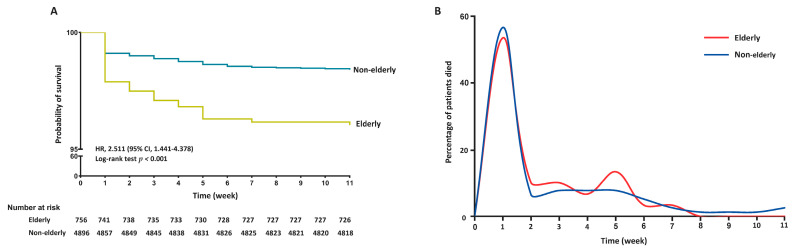
Characteristics of in-hospital survival rates (**A**) and mortality patterns over time (**B**) between elderly and non-elderly patients. HR, hazard ratio.

The two groups of patients presented distinct patterns of mortality over time. As illustrated in Figure 4A, the Kaplan–Meier survival curves indicated a significantly lower probability of survival in elderly patients compared with non-elderly patients, with a hazard ratio (HR) of 2.511 (95% CI: 1.441–4.378) and a log-rank test result (*p* < 0.001). As shown in Figure 4B, mortality rates of non-elderly patients presented a single peak within the first two weeks, followed by a steady decline after the second week. This pattern was consistent with a previous report [26,27]. In contrast, elderly patients with AP exhibited a biphasic pattern of mortality, with the first peak occurring within the first two weeks and the second peak occurring in the fifth week. In both groups, the proportion of deaths within the first week was not significantly different (53.33% vs. 56.41%, *p* > 0.05), indicating a high risk of mortality in the early stages. By the fifth week, the proportion of deaths in non-elderly patients declined to 7.69%, while it remained high at 13.33% in elderly patients.

## 4. Discussion

The findings of this cohort study indicate that elderly patients with AP suffered from more severe illness, with a higher incidence of comorbidities, organ failure, local complications, infection, and ICU stay. The increased mortality in elderly patients was strongly associated with POF, MOF, and infections. Notably, our data, for the first time, indicated that the deaths of elderly patients exhibited a biphasic mortality pattern, with the first peak within the first week and a second peak in the fifth week, whereas non-elderly patients exhibited a single peak in the first week.

In elderly patients, biliary etiology is the most common cause of AP, differing from HTG in non-elderly patients. Gallstone formation, delayed gallbladder emptying, and bile duct dilatation may contribute to the development of AP in aging adults [28], as reported in a previous study [29]. Additionally, elderly patients had higher CCI and more comorbidities, lower levels of albumin, and higher levels of interleukin 6 and creatinine at admission, indicating differences in inflammatory response and renal function [9,28].

The mortality rate of elderly patients with AP was approximately 2.9 times higher than that of non-elderly patients, which was consistent with previous studies [28,30] and a meta-analysis [31]. Meanwhile, the ratios of POF and MOF were 1.62 and 1.75 times greater than those of non-elderly patients, respectively, mainly involving respiratory and cardiovascular systems. The principal causes of the elevated mortality rates observed in the elderly were organ failures, particularly cardiovascular and renal complications [32]. Furthermore, our data showed higher susceptibility to infections among elderly patients with AP, which was not widely reported in other studies [10,11,33,34]. The presence of a CCI > 2, severe comorbidities [35], and multiple diseases may serve to exacerbate AP progression, thereby increasing the mortality rate [34,36,37]. Although we tracked those who had unplanned discharges, not all patients were followed up for the long term. Further analysis revealed that patients aged 60 to 70 years had significantly higher SIRS scores compared to those aged ≥ 80 years [1 (1–3) vs. 1 (0–2), *p* = 0.045]. This suggests a more intense systemic inflammatory response in this group. Elevated SIRS scores are typically associated with a higher risk of complications, such as MOF and POF, which may explain the higher mortality observed in patients aged 60 to 70 years [38,39]. We propose that patients in this age group, who are in the early stages of aging, retain a relatively stronger immune system compared to those aged ≥ 80. However, this more robust immune response may also lead to an exaggerated inflammatory response, increasing the risk of severe complications like MOF and POF, thereby contributing to the higher mortality observed. These clinical findings are corroborated by the results of animal studies [40,41], which demonstrated that aging exacerbates intestinal barrier dysfunction, bacterial translocation, and intestinal immunity in AP, thereby precipitating inflammation and organ injury.

The deaths of elderly patients increased over time during hospitalization, presenting a biphasic pattern. The data suggest that patients with AP suffer from organ failure due to a systemic inflammatory response in the early stage and peripancreatic infection in the late stage [22]. Although advances in IPN treatment have effectively eliminated the second mortality peak in non-elderly AP patients [27], late mortality of AP in elderly patients remains high. A recent study [42] demonstrated that the minimally invasive step-up approach reduces the morbidity of complications and pancreatic insufficiency in acute necrotizing pancreatitis. Therefore, it would be beneficial for future studies to explore whether this approach could improve the prognosis and reduce the complications of AP in elderly patients. In conclusion, comprehensive and individualized therapeutic strategies, including surveillance for organ function and the management of local complications and infections, should be utilized for elderly patients based on their risk stratifications.

There are several notable strengths of this study. Firstly, we confirmed that the clinical characteristics and outcomes of AP differ between elderly and younger patients. To the best of our knowledge, this is the first study revealing the different mortality patterns between elderly and non-elderly patients with AP. Secondly, this cohort study has a large sample size, comprising more than 5000 patients with AP over 6 years in a tertiary healthcare setting. This hospital is the leading medical center for AP in China with an average of over 2500 AP cases annually. Thirdly, the data were extracted from the electronic medical records, and the severity scores were assessed by research assistants and subsequently reviewed by experienced physicians. It must be acknowledged that this study is not without limitations. Firstly, despite the large sample size, the data were obtained from a single tertiary care hospital; consequently, the findings may not be generalizable to all elderly AP patients. Additionally, the follow-up period was limited, covering only outcomes during hospitalization and three months after discharge. Further studies are needed to assess the long-term survival and quality of life in elderly patients. Accordingly, as a single-center cohort study, the findings require further validation in multicenter prospective cohort studies.

## 5. Conclusions

Elderly patients with AP had different clinical characteristics and worse outcomes compared to young and middle-aged patients. The findings of this study highlight that elderly patients represent a significant challenge to further improving the prognosis of AP. It is imperative that greater attention be devoted to the optimization of individualized therapeutic strategies for elderly patients with AP.

## Figures and Tables

**Table 1:** Baseline characteristics of the patients.

Parameters	Elderly *n* = 756	Non-Elderly *n* = 4896	*p* Value
Age, years *	71 (66–81)	44 (36–51)	<0.001
Male, *n* (%)	361 (47.8)	3295 (67.3)	<0.001
Intervals from the onset to admission, hours *	24 (11–44)	24 (12–36)	0.909
CCI *	1 (0–1)	0 (0–1)	<0.001
Etiology, *n* (%)			<0.001
Hypertriglyceridemia	42 (5.6)	1933 (39.5)	
Biliary	417 (55.2)	943 (19.3)	
Alcohol	15 (2.0)	218 (4.5)	
Idiopathic	172 (22.8)	1073 (21.9)	
Mixed	10 (1.3)	223 (4.6)	
Others ‡	100 (13.2)	506 (10.3)	
Referral, *n* (%)	422 (55.8)	2633 (53.8)	0.294
Recurrent, *n* (%)	208 (27.5)	2326 (47.5)	<0.001
ASA classification, *n* (%)			<0.001
I	240 (31.7)	2409 (49.2)	
II	368 (48.7)	2269 (46.3)	
III	14 (1.9)	29 (0.6)	
IV	134 (17.7)	189 (3.9)	
Comorbidity, *n* (%)			
Diabetes mellites	60 (7.9)	360 (7.4)	0.569
Cardiovascular diseases	264 (34.9)	335 (6.8)	<0.001
Pulmonary diseases	61 (8.1)	22 (0.4)	<0.001
Renal diseases	12 (1.6)	12 (0.2)	<0.001
Neurological diseases	28 (3.7)	5 (0.1)	<0.001
Liver diseases	15 (2.0)	114 (2.3)	0.555
Admission laboratory markers *			
White blood cell counts, ×10^9^/L	12.76 (9.30–16.08)	13.12 (10.03–15.97)	0.077
Hematocrit, L/L	0.40 (0.37–0.44)	0.43 (0.39–0.46)	<0.001
Albumin, g/L	39.31 (35.70–43.10)	41.40 (37.80–45.30)	<0.001
Triglycerides, mmol/L	1.04 (0.73–1.68)	5.74 (1.65–13.10)	<0.001
Serum glucose, mmol/L	8.33 (6.58–10.76)	8.55 (6.37–11.69)	0.192
Blood urinary nitrogen, mmol/L	6.19 (4.90–8.30)	4.88 (3.60–6.12)	<0.001
Creatinine, mmol/L	79 (64–97)	73 (60–84)	<0.001
Calcium ions, mmol/L	2.16 (2.01–2.28)	2.18 (1.96–2.31)	0.382
C-reactive protein	118.7 (74.92–177.94)	125.71 (79.06–201.43)	0.001
Interleukin 6	438.07 (177.45–609.01)	349.34 (98.28–572.51)	<0.001
Admission severity scores *			
BISAP	2 (1–2)	1 (0–1)	<0.001
Glasgow	2 (1–3)	1 (0–2)	<0.001
SIRS	1 (1–2)	2 (1–3)	<0.001
SOFA	0 (0–1)	0 (0–1)	<0.001
MCTSI	0 (0–0)	1 (1–1)	0.001

* Values are median (IQR). Note. CCI, Charleson comorbidity index; ASA classification, American Analgesic Association classification; BISAP, bedside index for severity in acute pancreatitis; SIRS, systemic inflammatory response syndrome; SOFA, sequential organ failure assessment; MCTSI, Modified Computed Tomography Severity Index. ‡ Others included medication, traumatic, endoscopic retrograde cholangiopancreatography, auto-immune diseases, and other less common etiologies.

**Table 2:** Univariate analysis of clinical outcomes of patients.

Parameters	Full Cohort			Moderately Severe and Severe Acute Pancreatitis		
	Elderly *n* = 756	Non-Elderly *n* = 4896	*p* Value	Elderly *n* = 398	Non-Elderly *n* = 2493	*p* Value
Mortality, *n* (%)	30 (4.0)	78 (1.6)	<0.001	30 (7.5)	78 (3.1)	<0.001
Cause of death, *n* (%)						
Organ failure	14 (1.9)	25 (0.5)	<0.001	14 (3.5)	25 (1.0)	<0.001
Infection	2 (0.3)	6 (0.1)	0.655	2 (0.5)	6 (0.2)	0.684
Organ failure & Infection	13 (1.7)	41 (0.8)	0.034	13 (3.3)	41 (1.6)	0.026
Other	1 (0.1)	6 (0.1)	1.000	1 (0.3)	6 (0.2)	1.000
Length of hospital stay, d *	12 (8–16)	9 (7–14)	<0.001	14 (9–20)	12 (8–17)	<0.001
Hospital costs, ¥/person *	21,375.1 (12,482.8–34,138.5)	14,455.5 (8971.3–26,105.5)	<0.001	28,481.5 (17,111.8–49,404.8)	22,311.0 (13,417.0–38,730.0)	<0.001
Length of ICU stay, d *	0 (0–0)	0 (0–0)	0.040	0 (0–0)	0 (0–0)	0.117
ICU admission, *n* (%)	31 (4.1)	143 (2.9)	0.080	31 (7.8)	143 (5.7)	0.110
Local complication, *n* (%)						
APFC	259 (34.3)	2009 (41.0)	<0.001	259 (65.1)	2009 (80.6)	<0.001
ANC	50 (6.6)	489 (10.0)	0.003	50 (12.6)	489 (19.6)	0.001
IPN	13 (1.7)	67 (1.4)	0.447	13 (3.3)	67 (2.7)	0.513
Infection, *n* (%) ‡	189 (25.0)	581 (11.9)	<0.001	135 (33.9)	481 (19.3)	<0.001
Lung	150 (19.8)	420 (8.6)	<0.001	110 (27.6)	350 (14.0)	<0.001
Blood	64 (8.5)	206 (4.2)	<0.001	51 (12.8)	183 (7.3)	<0.001
Urinary	7 (0.9)	42 (0.9)	0.851	4 (1.0)	38 (1.5)	0.421
Abdominal drainage	7 (0.9)	67 (1.4)	0.410	7 (1.8)	62 (2.5)	0.377
Persistent organ failure, *n* (%)						
Respiratory	179 (23.7)	878 (17.9)	<0.001	179 (45.0)	878 (35.2)	<0.001
Cardiovascular	34 (4.5)	140 (2.9)	0.015	34 (8.5)	140 (5.6)	0.023
Renal	32 (4.2)	144 (2.9)	0.057	32 (4.2)	144 (2.9)	0.057
Duration of organ failure, d *						
Respiratory	0 (0–1)	0 (0–1)	<0.001	1 (0–4.3)	1 (0–4)	<0.001
Cardiovascular	0 (0–0)	0 (0–0)	0.006	0 (0–0)	0 (0–0)	0.009
Renal	0 (0–0)	0 (0–0)	0.005	0 (0–0)	0 (0–0)	0.008
Organ supportive care, *n* (%)						
NIPPV	130 (17.2)	668 (13.6)	0.009	130 (32.7)	668 (26.8)	0.015
IMV	50 (6.6)	230 (4.7)	0.024	50 (12.6)	230 (9.2)	0.037
CRRT	16 (2.1)	69 (1.4)	0.137	16 (4.0)	69 (2.8)	0.170
Severity, *n* (%)			<0.001			<0.001
Mild	358 (47.4)	2403 (49.1)				
Moderately severe	206 (27.2)	1586 (32.4)		206 (51.8)	1586 (63.6)	
Severe	192 (25.4)	907 (18.5)		192 (48.2)	907 (36.4)	
Necrosectomy, *n* (%)	23 (3.0)	163 (3.3)	0.681	21 (5.3)	148 (5.9)	0.602
Cholecystectomy, *n* (%)	80 (10.6)	216 (4.4)	<0.001	38 (9.5)	75 (3.0)	<0.001

* Values are median (IQR). ‡ Infection was defined by microbiological diagnosis from the sample of sputum, pleural fluid, blood, urine, and drainage of abdominal. Note. ICU, intensive care unit; APFC, acute peripancreatic fluid collections; ANC, acute necrotic collections; IPN, infected pancreatic necrosis; NIPPV, non-invasive positive pressure ventilation; IMV, intermittent mandatory ventilation; CRRT, Continuous Renal Replacement Therapy.

**Table 3:** Adjusted *p* value for clinical outcomes.

Parameters	Elderly *n* = 756	Non-Elderly *n* = 4896	Crude OR (95% CI)	Crude *p* Value	Adjusted OR (95% CI) †	Adjusted *p* Value
Mortality, *n* (%)	30 (4.0)	78 (1.6)	2.552 (1.663–3.917)	<0.001	2.911 (1.801–4.706)	<0.001
ICU admission, *n* (%)	31 (4.1)	143 (2.9)	1.421 (0.956–2.112)	0.080	1.739 (1.126–2.685)	0.013
Persistent organ failure, *n* (%)	192 (25.4)	907 (18.5)	1.497 (1.252–1.791)	<0.001	1.623 (1.326–1.987)	<0.001
Multiple organ failure, *n* (%)	39 (5.2)	184 (3.8)	1.393 (0.977–1.985)	0.066	1.757 (1.186–2.604)	0.005
Infection, *n* (%)	189 (25.0)	581 (11.9)	2.476 (2.055–2.982)	<0.001	2.451 (1.994–3.013)	<0.001

† Logistic regression with OR (95% CI) after adjusting baseline factors that each was with important clinical significance. These factors included the following: gender, CCI, comorbidity, referral status, onset hours, and admission triglyceride levels. Note: ICU, intensive care unit; CCI, Charleson comorbidity index.

**Table 4:** Multivariate logistic regression of influencing factors for mortality, ICU admission, infection, MOF, and POF in patients with acute pancreatitis.

Variables	Mortality		ICU Admission		Infection	
	OR (95% CI)	*p* Value	OR (95% CI)	*p* Value	OR (95% CI)	*p* Value
Age (≥60 vs. <60 years old)	2.911 (1.801–4.706)	<0.001	1.739 (1.126–2.685)	0.013	2.451 (1.994–3.013)	<0.001
Sex (male vs. female)	0.711 (0.479–1.056)	0.091	0.705 (0.514–0.966)	0.029	1.020 (0.864–1.203)	0.817
CCI (>2 vs. ≤2)	2.247 (1.045–4.831)	0.038	0.980 (0.420–2.285)	0.962	1.474 (1.012–2.148)	0.043
Comorbidity (yes vs. no)	0.788 (0.505–1.231)	0.296	0.931 (0.657–1.318)	0.686	1.025 (0.859–1.223)	0.784
Referral status (yes vs. no)	5.930 (3.319–10.598)	<0.001	5.071 (3.244–7.925)	<0.001	2.697 (2.247–3.237)	<0.001
Onset hours (≥24 vs. <24 h)	1.068 (0.695–1.643)	0.763	1.567 (1.090–2.251)	0.015	1.571 (1.314–1.879)	<0.001
Admission HTG (≥500 vs. <500 mg/dL)	1.733 (1.135–2.647)	0.011	2.038 (1.463–2.837)	<0.001	0.986 (0.831–1.169)	0.869
Variables	MOF				POF	
	OR (95% CI)	*p* Value			OR (95% CI)	*p* Value
Age (≥60 vs. <60 years old)	1.757 (1.186–2.604)	0.005			1.623 (1.326–1.987)	<0.001
Sex (male vs. female)	0.876 (0.658–1.167)	0.365			0.964 (0.832–1.116)	0.622
CCI (>2 vs. ≤2)	1.991 (1.126–3.522)	0.018			1.640 (1.162–2.313)	0.005
Comorbidity (yes vs. no)	1.116 (0.828–1.505)	0.471			1.208 (1.035–1.409)	0.017
Referral status (yes vs. no)	4.771 (3.233–7.039)	<0.001			4.570 (3.853–5.421)	<0.001
Onset hours (≥24 vs. <24 h)	1.915 (1.370–2.675)	<0.001			1.693 (1.446–1.983)	<0.001
Admission HTG (≥500 vs. <500 mg/dL)	2.279 (1.692–3.069)	<0.001			1.430 (1.233–1.659)	<0.001

Note. ICU, intensive care unit; MOF, multiple organ failure; POF, persistent organ failure; CCI, Charleson comorbidity index; HTG, hypertriglyceridemia.

## Data Availability

The data presented in this study are available on request from the corresponding author due to ethics and privacy reasons.
